# Kinematics and observer-animator kinematic similarity predict mental state attribution from Heider–Simmel style animations

**DOI:** 10.1038/s41598-021-97660-2

**Published:** 2021-09-14

**Authors:** Bianca A. Schuster, Dagmar S. Fraser, Jasper J. F. van den Bosch, Sophie Sowden, Andrew S. Gordon, Dongsung Huh, Jennifer L. Cook

**Affiliations:** 1 School of Psychology, University of Birmingham, Hills Building, Edgbaston Park Rd, Birmingham, B15 2TT UK; 2 Institute for Creative Technologies, University of Southern California, Los Angeles, CA 90094 USA; 3 MIT-IBM Watson AI Lab, Cambridge, MA 02142 USA

**Keywords:** Psychology, Human behaviour, Huntington's disease, Movement disorders, Neurodevelopmental disorders, Parkinson's disease, Cognitive neuroscience, Motor control, Social behaviour, Social neuroscience

## Abstract

The ability to ascribe mental states, such as beliefs or desires to oneself and other individuals forms an integral part of everyday social interaction. Animations tasks, in which observers watch videos of interacting triangles, have been extensively used to test mental state attribution in a variety of clinical populations. Compared to control participants, individuals with clinical conditions such as autism typically offer less appropriate mental state descriptions of such videos. Recent research suggests that stimulus kinematics and movement similarity (between the video and the observer) may contribute to mental state attribution difficulties. Here we present a novel adaptation of the animations task, suitable to track and compare animation generator and -observer kinematics. Using this task and a population-derived stimulus database, we confirmed the hypotheses that an animation’s jerk and jerk similarity between observer and animator significantly contribute to the correct identification of an animation. By employing random forest analysis to explore other stimulus characteristics, we reveal that other indices of movement similarity, including acceleration- and rotation-based similarity, also predict performance. Our results highlight the importance of movement similarity between observer and animator and raise new questions about reasons why some clinical populations exhibit difficulties with this task.

## Introduction

Seminal work by Heider and Simmel^1^ demonstrated that humans readily attribute mental states to two triangles moving around a rectangular enclosure. Since their inception in 1944 such “animations tasks” (also referred to as Frith-Happé Animations^2^ and Social Attribution Task^3^) have grown dramatically in popularity and have been used in a wide variety of clinical populations, including autism spectrum disorder (ASD)^2,4^, schizophrenia^5^, antisocial personality disorder^6^, Huntington’s disease^7^ and Tourette’s syndrome^8^. Though animations tasks have been scored and administered in a number of ways (some studies count the number of mental state terms used to describe the movements of the triangles^2,4^, other studies have asked participants to rate the type of interaction or the mental state word depicted in the animations^9,10^) it is generally agreed that “poor performance” indicates a problem with ascribing appropriate mental states to the triangles. We refer to this process here as ‘mental state attribution’.

Though mental state attribution has been found to be atypical across a range of clinical populations, little is known about *why* some individuals struggle to attribute appropriate mental states to the triangles. One explanation is that individuals who struggle with the animations task would exhibit atypicalities in other tests of mental state attribution because of a deficit in the ability to attribute minds and ascribe appropriate mental states (i.e., Theory of Mind [ToM]). However, animations tasks tend to be more sensitive to mental state attribution difficulties compared to other tests, as shown by Abell et al.^2^. At present it is unclear why some individuals find this task particularly challenging.

A recent study highlights that kinematic similarities between the triangles’ movements and the participant’s own movements may influence performance in the animations task^9^. Edey and colleagues asked autistic (‘condition-first’ terminology is used in line with the majority preference expressed in a survey of the autistic community^11^) and non-autistic participants to complete the animations task, and also to produce their own animations using triangles that could be moved around an enclosure with magnetic levers. In line with a growing literature concerning jerky body movements in autism^12–15^, the authors found that animations produced by autistic individuals were more jerky (i.e., exhibited greater changes in acceleration and deceleration) than those produced by non-autistic individuals. Furthermore, whereas non-autistic participants could readily attribute mental states to animations created by other non-autistic participants, they had difficulties attributing mental states to the jerky animations that had been produced by the autistic participants. Conversely, autistic participants in Edey’s study did not show improved performance when rating their own group’s, relative to the control group’s, animations. The authors proposed that *jerk similarity* significantly contributes to performance in the animations task: that is, non-autistic individuals were better able to correctly identify animations created by other non-autistic participants because the kinematic jerk in the videos was comparable to the amount of jerk they themselves would exhibit when moving the triangles. Autistic participants did not, however, benefit from jerk similarity because high variability in jerk present within this group led to a reduced number of animations sufficiently similar to facilitate mentalizing performance.

Although Edey et al.^9^ inferred—on the basis that jerk differed between the ASD and control group—that jerk similarity was a likely contributor to animations task performance, they did not empirically demonstrate this to be the case. To test Edey et al.’s, hypothesis and better understand why some individuals struggle to attribute appropriate mental states in the animations task, the first aim of the current study was to test whether a significant amount of variance in performance in a Heider–Simmel style animations task would be accounted for by the kinematic jerkiness of the animation and the *similarity* between the jerkiness of the animation and a participant’s own movements.

Kinematic jerk and jerk similarity are not the only factors which plausibly influence performance in animations tasks. Previous studies have highlighted potential roles for stimulus features including the rotation of, and distance between, the triangles^16^, and the shape of the triangles’ trajectories^17^. For instance, Roux et al. documented highly distinguishable trajectory paths for random, goal-directed and mental state animations, thus suggesting that trajectory path may be an important cue that observers can use to attribute appropriate mental states. Furthermore, it is plausible that, in addition to jerk, other kinematic parameters such as speed and acceleration may predict performance. That is, beyond jerk similarity, *movement similarity* more generally may predict the accuracy of mental state attribution in the animations task. The proposal that movement similarity may affect performance in the animations task is bolstered by recent empirical work showing that observers more accurately estimate a human actor’s underlying intentions when the trajectory of the actor’s arm movements closely approximates the observer’s own movements^18^. Furthermore, a role for movement similarity in mental state attribution is in line with theoretical accounts suggesting that inferences about others’ actions are facilitated by mapping visual representations of these actions onto our own visual/motoric representations of the same actions^19–22^, and is broadly consistent with simulation accounts of ToM which claim that one uses one's own mental processes to simulate others’ mental states^23^. The movement similarity hypothesis would propose that mental state attribution difficulties in classic animations tasks may, at least in part, be explained by differences between the way the triangles are animated and the way an observer would move the triangles if required to create their own animation.

Correspondingly, the second aim of the current study was to explore the extent to which a range of other stimulus features (including trajectory shape, rotation of and distance between the triangles, and various indices of kinematics) influence the ease with which participants correctly attribute a mental state to an animation. By doing so, we shed light on a multiplicity of factors which may explain why some clinical groups find the animations task so challenging.

For this latter analysis we made use of the fact that, similar to a sound wave, a triangle’s trajectory comprises a complex wave and thus can be decomposed with Fourier transform and represented as spectral density (i.e., energy) in different frequency bands^24^. In other words, Fourier transform can be used to characterize the shape of a trajectory. For example, a trajectory which approximately follows an elliptical orbit oscillates in speed and curvature twice during every full rotation and consequently would be characterized by high spectral density in a band centered around an angular frequency of two. Adapting a method developed by Huh and Sejnowski, we explored whether there are particular angular frequency bands which differentiate mocking, seducing, surprising, following and fighting animations and whether spectral density in these bands is predictive of accuracy.

**Figure 1:**
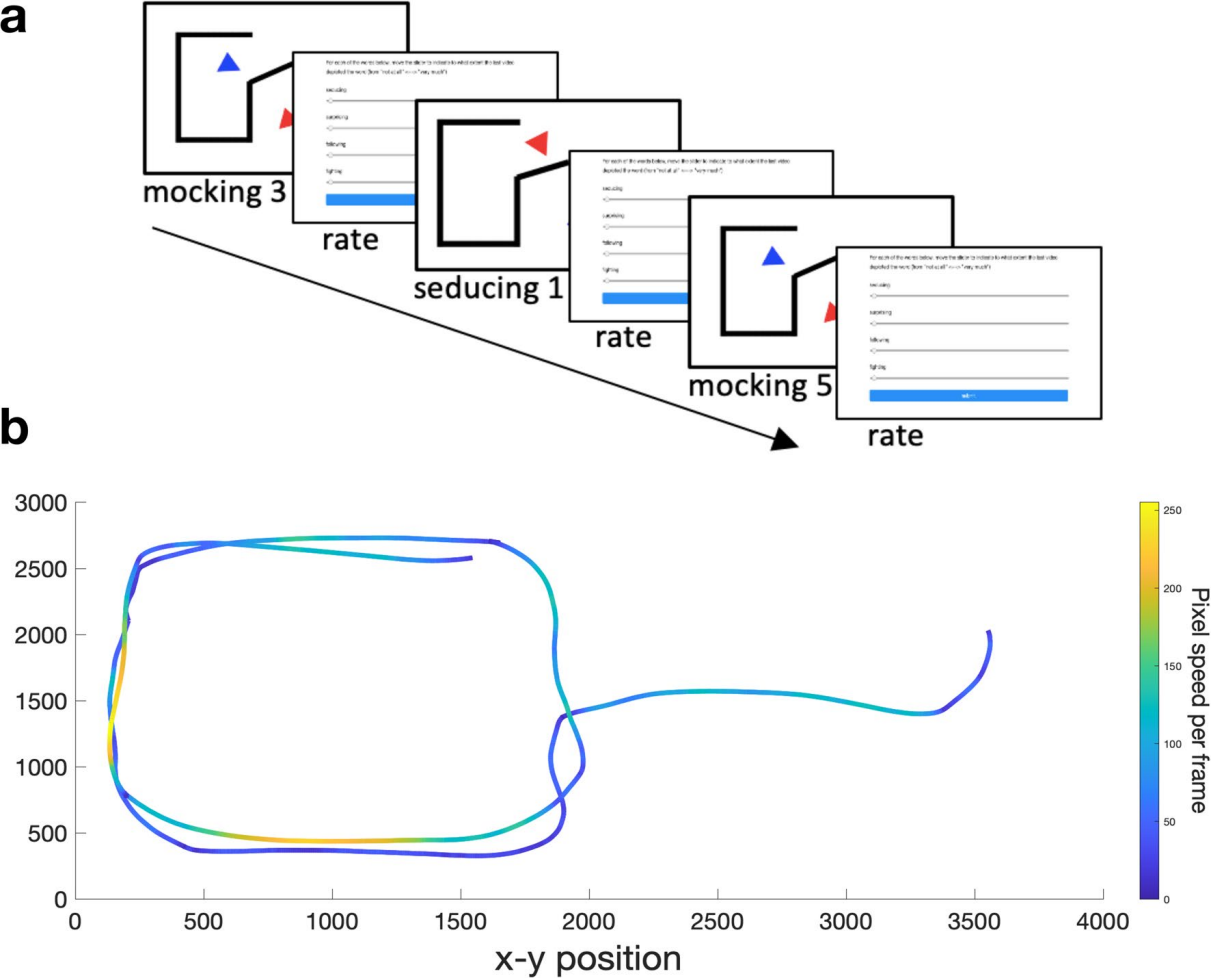
(**a**) Schematic depiction of three successive trials in the animations task*.* 37 participants watched videos from the database and rated the extent to which each video depicted mocking, seducing, surprising, following, or fighting. (**b**) Example trajectory of an animation stimulus. Each participant used a touchscreen device to create their own triangles animations. For each animation (both observed and generated by participants) we calculated *jerk* as the mean of the third order non-null derivative of the raw positional data across all frames; jerk similarity was calculated as the difference in mean jerk between an animation stimulus and the participant’s own animation of the same word (*jerk difference*). Depicted is an example of a *following* animation (one triangle’s trajectory).

Currently available animations task stimulus sets are not suitable to test our hypotheses regarding jerk, jerk similarity and movement similarity for two reasons: First, having been created by experimenters or graphic designers, the stimuli in these tasks typically represent a narrow range of kinematics and thus lack the variation necessary for quantifying the contribution of kinematics and other stimulus features to performance. Second, tasks to date offer no option to track animator (or observer) kinematics at sufficient sampling rates to reliably make inferences about the role of jerk/movement similarity. Here we created a novel Heider–Simmel style animations database (available upon request) by asking 51 members of the general population to animate two triangles to depict mental (mocking, seducing, surprising) and non-mental- (following, fighting) state interactions on a 133 Hz touch screen device. The distinction between mental and non-mental states, and the choice of individual words used within these conditions, was based on the ToM and Goal-Directed conditions used in the original Frith-Happé animations^2^. That is, ToM animations depict “[…] one character reacting to the other character’s mental state […]” whereas Goal-Directed animations represent “[…] reciprocal interaction, but no implication that one character was reading the other's ‘mind’” (p. 5). This distinction has since been widely used across the literature^4,9,10^. Following database creation, an independent sample of 37 members of the general population watched a selection of videos from our new database. To ensure that participants were exposed to a wide range of kinematics, they watched 8 exemplars for each word, ranging from slow to fast speed. Participants rated the extent to which each animation depicted the words mocking, seducing, surprising, following and fighting, in addition to also creating their own animation for each word (Fig. 1). In a four-step analysis procedure, we first used Bayesian mixed effects models to test our hypotheses that kinematic jerk and the similarity in jerk between observer and animator are significant predictors of the accuracy of mental state attributions (*confirmatory analysis*). In a second step, we used Fast Fourier Transform (FFT) combined with bootstrapped F-tests to investigate whether mocking, seducing, surprising, following and fighting animations could be reliably distinguished according to their profile of spectral density across a range of frequency bands (*exploratory analysis 1*) thus enabling us to identify potential differences in trajectory shape between animations of different words. In a further exploratory analysis, we employed a random forest procedure to determine the relative contribution to accuracy of a multiplicity of factors including speed, acceleration, jerk, the amount of simultaneous movement of both triangles, the relative distance between triangles, triangles’ average rotation and trajectory shape, as indexed by the magnitude of spectral density in the frequency bands identified in the second analysis step (*exploratory analysis 2*). Finally, in *exploratory analysis 3* we took the top three predictors from exploratory analysis 2 and used these to calculate novel indices of movement similarity; Bayesian mixed effects models were employed to test whether these novel indices are significant predictors of animations task performance.

## Results

Accuracy for each trial was calculated by subtracting the mean rating for all non-target words from the rating for the target word (e.g., the target word was *seducing* on trials where the participant watched a video wherein the original animator had attempted to depict the triangles seducing each other). Consequently, a high, positive accuracy score for a seducing animation indicates that an observer rated this animation as depicting seducing to a higher extent than mocking, surprising, following or fighting. For a comparison of mean accuracy scores for each word category see Supplementary Materials. For each video that participants observed and for each animation that they created themselves, mean jerk magnitude (hereafter: *jerk*) was obtained by taking the third order non-null derivatives of the raw positional data and calculating the mean across all frames in the video. Jerk similarity was calculated as the difference in mean jerk between an animation stimulus and the participant’s own animation of the same word (hereafter: *jerk difference*), where lower difference values indicate higher jerk similarity (see Methods: Data Analysis and Processing).

### Mental state animations are rated less accurately than non-mental state animations

A Bayesian linear mixed effects model with the maximal random effects structure allowed by the design^25^ was fitted to jerk, jerk difference (lower values reflect higher jerk similarity) and the dummy-coded factor *mental state* (mental state [seducing, surprising, mocking] versus non-mental state [following, fighting]) as well as their three-way interaction. Random intercepts were fitted for *animation ID* (unique identifier for each animation) and *subject ID;* random slopes were fitted for the interaction between jerk and mental state varying by *animation ID* and jerk difference varying by *subject ID*. For all relevant model parameters, we report expected values ($E_{\mu }$) under the posterior distribution and their 95% credible intervals (CrIs)^26^, as well as the posterior probability that an effect is different to zero (*P* $(E_{\mu } < 0)$/*P* $(E_{\mu } > 0)$). In line with Franke and Roettger^27^, if a hypothesis states that an effect $E_{\mu } \ne 0$ (e.g. effect of jerk similarity on accuracy), we conclude there is compelling evidence for this effect if zero is not included in the 95% CrI of $E_{\mu }$ and if the posterior probability P($E_{\mu } \ne 0$) is close to 1.

**Figure 2:**
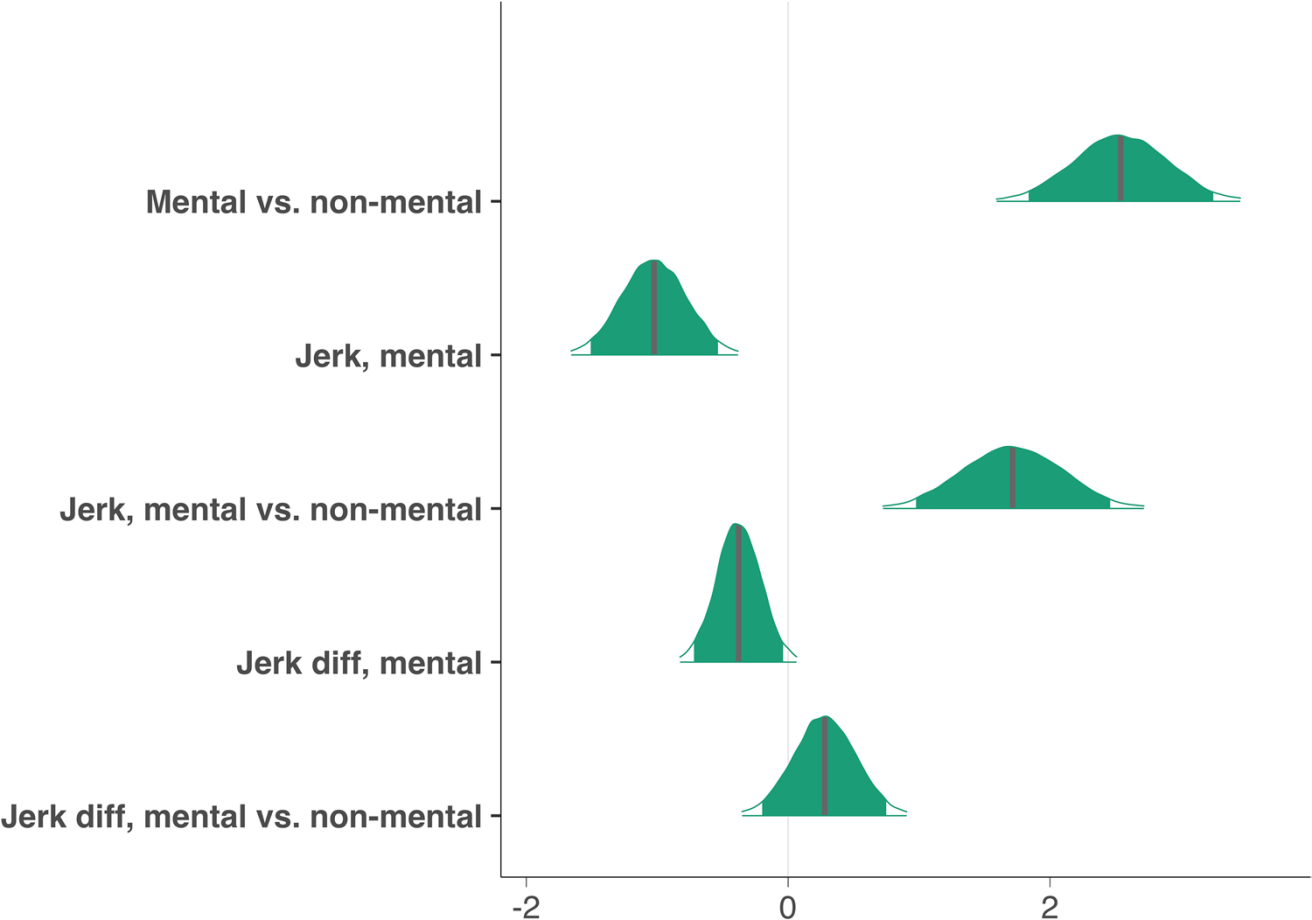
Posterior probabilities of model parameters predicting accuracy. Filled green areas represent 95% credible Intervals around parameter estimates. Grey lines represent means of parameter estimates. ‘Jerk diff’ = jerk difference.

The model indicated that accuracy was higher in non-mental state videos relative to mental state videos ($E\mu_{mentalVSnon - mental}$ = 2.54, CrI = [1.81, 3.28]), with the posterior probability that the difference is larger than zero being P $(E\mu_{mentalVSnon - mental} > 0)$ = 1 (see Fig. 2 for prior and posterior distributions of all estimated parameters).

### Jerk affects performance differently for mental- and non-mental state animations

In line with our hypothesis, accuracy was associated with mean jerk, furthermore jerk interacted with mental state: For mental state animations, lower mean jerk was associated with higher accuracy ($E\mu_{jerk,mental}$ =  − 1.03, CrI = [− 1.52, − 0.53]), whereas in non-mental state animations higher mean jerk led to higher accuracy scores ($E\mu_{jerk,mentalVSnon - mental}$ = 1.65, CrI = [0.88, 2.41]). Thus, while mental state animations with mean jerk values 1 standard deviation (SD) above the (mental state condition) mean were rated 1.03 points less accurately (compared to a mental state video with a mean jerk value), non-mental state animations with jerk values 1 SD above the (mental state condition) mean were rated 0.62 points more accurately ($E\mu_{jerk,mental} + E\mu_{jerk,mentalVSnon - mental}$ =  − 1.03 + 1.65). Since the posterior probabilities for both effects (P($E\mu_{jerk,mentalVSnon - mental} > 0)$, P($E\mu_{jerk,mental} < 0$)) were in fact 1, we conclude that, given our model and the data, there is compelling evidence in favor of our hypothesis that an animations’ jerk impacts mental state attribution performance in the animations task. To probe whether such effects varied as a function of the word depicted in the video, we conducted separate exploratory models for non-mental state and mental state animations for which we included *word category* (non-mental state: following, fighting; mental state: mocking, seducing, surprising) as a predictor in addition to jerk and jerk difference. These models revealed that, for non-mental state animations there was a strong positive effect of jerk for fighting, but not following, animations ($E\mu_{jerk,fighting}$ = 1.88, CrI = [0.67, 3.11], P $(E\mu_{jerk,fighting} > 0)$ = 1; $E\mu_{jerk,following}$ = 0.30, CrI = [− 0.30, 1.05]). For mental state animations, the overall negative effect of jerk was driven by a tendency towards a negative effect of jerk on accuracy in mocking and surprising animations ($E\mu_{jerk,mocking}$ =  − 0.58, CrI = [− 1.56, 0.40]; $E\mu_{jerk,surprising}$ =  − 0.94, CrI = [− 2.69, 0.76]). There was no effect of jerk in seducing animations ($E\mu_{jerk,seducing}$ = 0.26, CrI = [− 1.40, 1.85]).

### Higher observer-animator similarity in jerk is associated with higher accuracy

In line with our hypothesis, accuracy was also associated with jerk difference. The model revealed a negative relationship between jerk difference and accuracy for mental state animations ($E\mu_{jerkDiff,mental}$ =  − 0.38, CrI = [− 0.72, − 0.03]; P($E\mu_{jerkDiff,mental}$ < 0) = 0.98). Jerk difference did not affect accuracy differently in non-mental state animations, indicated by high uncertainty surrounding the coefficient for the contrast of mental state and non-mental state ($E\mu_{jerkDiff,mentalVSnon - mental}$ = 0.25, CrI = [− 0.27, 0.76]). Thus, jerk difference had a negative effect on accuracy in both mental- and non-mental state animations. Consequently, higher jerk similarity was associated with higher accuracy. To probe whether such effects varied as a function of word category we conducted an exploratory mixed model which included the word categories mocking, seducing and surprising. This model revealed that the negative main effect of jerk difference was mainly driven by mocking animations ($E\mu_{jerkDiff,mocking}$ =  − 0.70, CrI = [− 1.22, − 0.18]; P ($E\mu_{jerkDiff,mocking} < 0$) = 0.99; $E\mu_{jerkDiff,seducing}$ = 0.98, CrI = [− 0.49, 2.46]; $E\mu_{jerkDiff,surprising}$ = 0.63, CrI = [− 0.29, 1.52]).

### A combination of ten kinematic and spatial variables best predicts accuracy in the animations task

**Figure 3:**
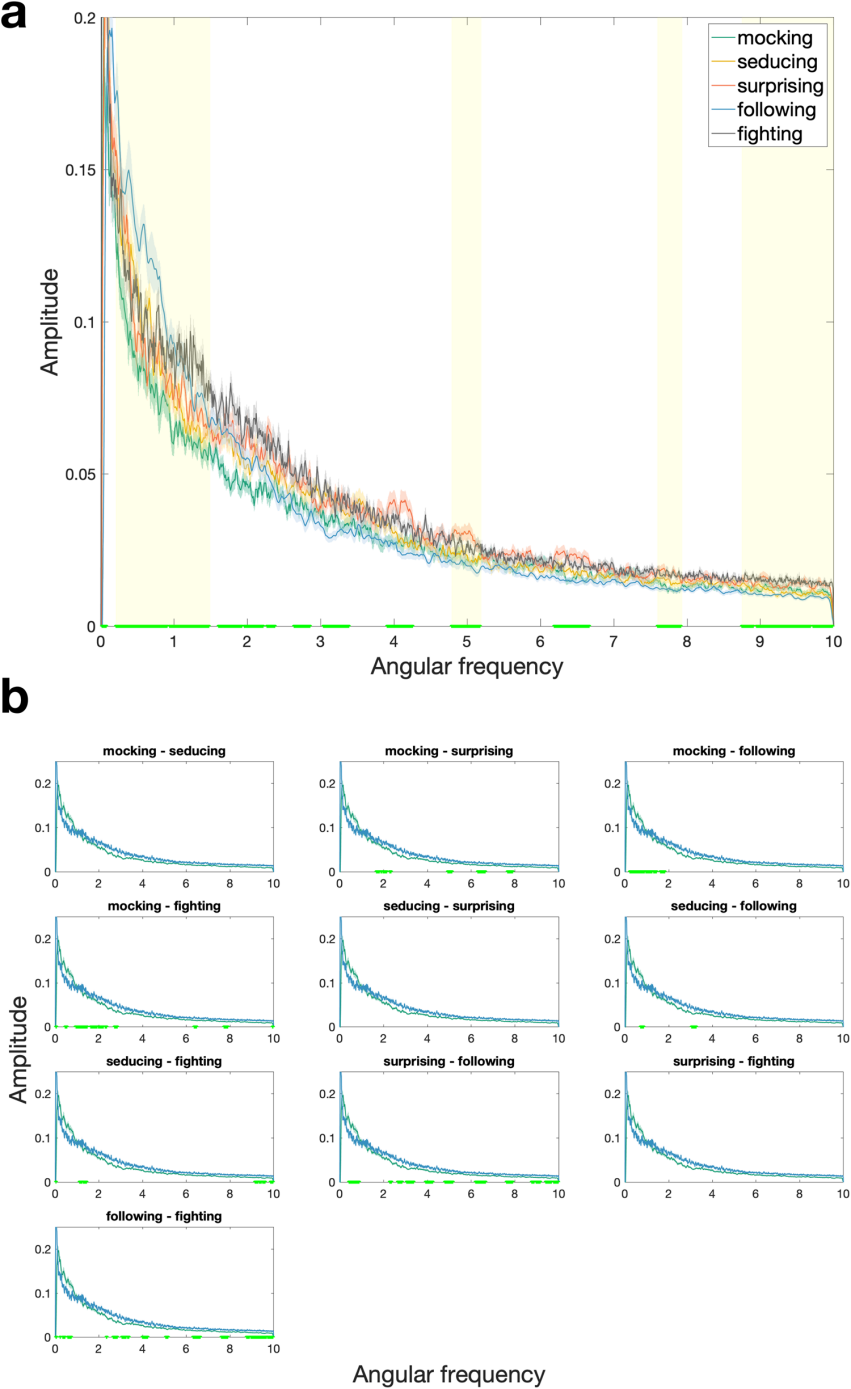
(**a**) Significant clusters of difference in angular frequency spectral density (AFSD). Solid colored lines represent spectral density as a function of angular frequency per word (i.e., AFSD), the corresponding shaded areas represent 1 SEM (standard error of the mean) below and above the mean values. Yellow bars on x-axis represent clusters where AFSD significantly differs between mocking, seducing, surprising, following and fighting. Clusters that are predictive of accuracy are highlighted in yellow. Note that the lowest angular frequency derived from the data varied between 0.02 and 0.09, resulting in extrapolated values for some participants. For this reason, the first cluster of difference ranging from 0.02 to 0.09 was considered not representative of actual movements and disregarded. (**b**) Post-hoc comparisons of AFSD.

In order to explore the relative importance of trajectory path alongside a variety of other stimulus features, we first identified which components of triangle trajectories can reliably distinguish between the five target words (i.e., mocking, seducing, surprising, following, fighting). For this we used FFT to decompose the triangles’ trajectories and represent them as an amplitude spectral density profile across a range of angular frequencies. We then employed bootstrapped F-tests (with 1000 boots) to identify angular frequency “bins” wherein spectral density significantly differs between the five target words (see Methods: Data Analysis and Processing). We reasoned that these bins contain signal that participants may be using in the mental state attribution task. This analysis revealed nine significant clusters, defined as clusters of difference that occurred in less than 5% of comparisons with resampled distributions (see Fig. 3a).

To examine whether spectral density in these nine frequency clusters was predictive of accuracy we used the maxima and minima of each significant cluster as bin edges and calculated the *angular frequency spectral density (AFSD)* as the area under the curve between the bin edges (cluster bin edges: 0.21–1.49, 1.61–2.39, 2.64–2.87, 3.04–3.40, 3.91–4.27, 4.79–5.19, 6.19–6.68, 7.6–7.93, 8.75–10). AFSD in the nine bins refers to the relative amount of specific trajectory components (i.e., “shapes”) present in each animation. That is, an animation that predominantly features elliptical trajectory components would be characterized by high AFSD in a band centered around an angular frequency of two (i.e., bin 2 which covers angular frequencies from 1.61 to 2.39).

The relative contribution to accuracy of the presence of these trajectory components was then assessed, alongside a selection of other kinematic and spatial variables, which were chosen based on indications in previous literature for their putative role in mental state attribution^16,17^. For this purpose, mental-state, speed, acceleration magnitude (hereafter: *acceleration*), jerk, *simultaneous movement*, *relative distance* and *mean rotation* were entered into a random forest model^28^ using the *Boruta*^29^ wrapper algorithm (version 7.7.0). Boruta trains a random forest regression model on all variables as well as their permuted copies—so called “shadow features”—and classes a variable as *important* when its permutation importance is significantly higher than the highest permutation importance of a shadow feature (for more details see Methods: Exploratory analysis). Note that because this analysis technique does not account for random effects, values corresponding to the same animation were averaged across participants, this permits indices such as jerk and acceleration which are features of a particular animation but excludes jerk difference which depends on the relation between an animation and an individual participant.

**Figure 4:**
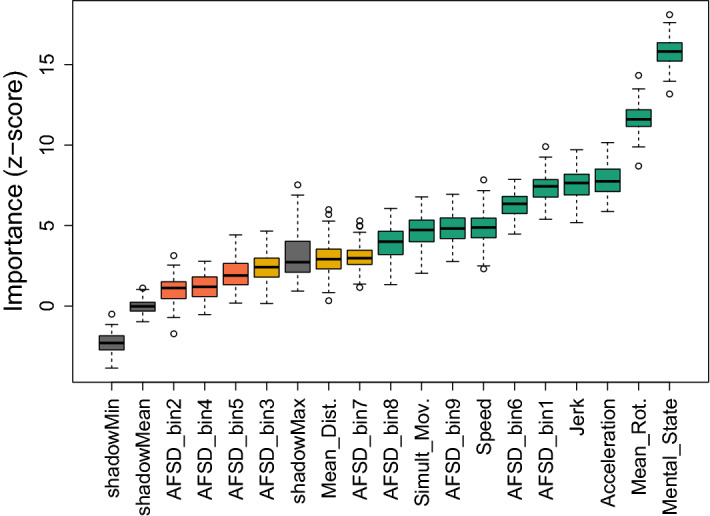
Random forest variable importances. Variable importances of all 16 features entered into the Boruta random forest, displayed as boxplots. Box edges denote the interquartile range (IQR) between first and third quartile; whiskers denote 1.5 * IQR distance from box edges; circles represent outliers outside of 1.5 * IQR above and below box edges. Box color denotes decision: Green = confirmed, yellow = tentative, red = rejected; grey = meta-attributes shadowMin, shadowMax and shadowMean (minimum, maximum and mean variable importance attained by a shadow feature).

Out of all 16 variables tested, 10 were confirmed *important*, two were confirmed *unimportant*, and four were classed as *tentative* on the basis that their permutation importance was not significantly different from the maximal importance of a shadow feature (see Fig. 4). Figure 4 illustrates that the important role of mental-state and jerk in predicting accuracy is confirmed by the random forest model, with mean importances of 16.0 and 7.82 respectively. However, the model identifies a third variable as even more important than jerk: mean rotation (mean importance = 11.78). In addition, an animation’s acceleration and speed, AFSD in bins 1, 6, 9 and 8, as well as the amount of simultaneous movement of both triangles notably contribute to explaining performance in the animations task (mean importances: acceleration = 7.91; speed = 4.70; AFSD-bin 1 = 7.03, AFSD-bin 6 = 6.37, AFSD-bin 9 = 5.04, AFSD-bin 8 = 3.89; simultaneous movement = 4.74). A final model of all 10 important variables predicting accuracy was evaluated by training a random forest on a subset of 70% of the data (training set) and using it to predict the remaining 30% (test set). The regression model of the training set predicting the test set was highly significant (*p* < 0.001) and indicated that the selected variables explained 37% of accuracy values.

We subsequently conducted post hoc random forests separately for mental state- and non-mental state animations. These post hoc analyses revealed that, in mental state animations, five factors were predictive of accuracy, with jerk and acceleration being the most prominent predictors, followed by speed, which was ranked third (see Supplementary Fig. 2). In addition, AFSD in bin 6 and simultaneous movement were classed as important in predicting accuracy. In non-mental state animations, a total of eight predictors were identified as important variables, with mean rotation being ranked highest by a considerable margin. In addition to mean rotation, a combination of AFSD in bins 1, 6, 7 and 9, and acceleration, jerk and speed were identified as important features of non-mental state animations.

### Similarity measures calculated from the three variables identified as most important in the random forest predict mental state attribution accuracy

In order to assess whether the effect of movement similarity between animator and observer on successful mental state attribution extends beyond jerk, we calculated similarity measures for the two variables (acceleration and rotation) which were identified as most important in the random forest analysis alongside jerk. Subsequently we employed two Bayesian mixed effects models to assess the strength of these difference scores in predicting accuracy. The first featured acceleration difference, rotation difference and mental state (dummy-coded) as predictors, and the second featured rotation difference, jerk difference and mental state. Model 1 revealed a negative effect for rotation difference in mental state animations ($E\mu_{rotationDiff, mental}$ =  − 0.34, CrI = [− 0.68, − 0.01]; P($E\mu_{rotationDiff}$ < 0) = 0.98), and this relationship did not differ for non-mental state animations ($E\mu_{rotationDiff, mentalVSnon - mental}$ = 0.40, CrI = [− 0.13, 0.93]) indicating that overall, higher rotation similarity was associated with higher accuracy. For acceleration difference, the model revealed an interaction with mental state, where acceleration difference was negatively associated with accuracy in mental state ($E\mu_{accelerationDiff, mental}$ =  − 0.48, CrI = [− 0.88, − 0.09]), but not related to accuracy in non-mental state animations ($E\mu_{accelerationDiff,mentalVSnon - mental}$ = 0.53, CrI = [0.01, 1.05]; note that this coefficient represents the *difference* in coefficients for acceleration difference between mental and non-mental state animations, the coefficient for the relationship between acceleration difference and accuracy in non-mental state animations is close to zero: 0.53–0.48 = 0.05). The second model revealed comparable results ($E\mu_{rotationDiff,mental}$ =  − 0.35, CrI = [− 0.66, − 0.04]; $E\mu_{rotationDiff,mentalVSnon - mental}$ = 0.35, CrI = [− 0.14, 0.85]; $E\mu_{jerkDiff, mental}$ =  − 0.48, CrI = [− 0.85, − 0.11]; $E\mu_{jerkDiff,mentalVSnon - mental}$ = 0.58, CrI = [0.09, 1.06]).

## Discussion

This study evaluated the relative contribution of jerk, jerk similarity and other stimulus characteristics to mental state attribution performance, indexed using a novel adaptation of the animations task, suitable to track and compare animation generator and -observer kinematics. Our results confirm our hypothesis that animation jerk and jerk similarity are predictors of the accuracy of mental state attribution. In addition, we highlight that stimulus features including the shape of the triangles’ trajectories and the amount of rotation of the triangles can also affect the ease with which participants are able to appropriately label the target states (*exploratory analysis 2*). Finally, we show that the similarity between an observer and generator is also beneficial when considering other movement characteristics beyond jerk, such as triangle rotation and acceleration (*exploratory analysis 3*).

In the first part of our confirmatory analysis step, we found that mental state was the primary predictor of animations task performance. Mental state videos were strongly associated with lower accuracy, correspondingly non-mental state videos were rated more accurately. The extant literature is mixed and there are some studies in which mental state animations are rated less accurately than non-mental state animations^10,30^. However, our observation that our healthy participants performed worse when interpreting mental, relative to non-mental, state animations, is inconsistent with most previous findings: In Abell et al.’s and other studies, non-autistic adult participants performed at least equally well^2,4,30–32^ on mental state and non-mental state animations. It is possible that our findings illustrate a true difference in difficulty between mental and non-mental state attribution that is revealed only when participants are presented with a wide range of animation stimuli from a population-derived database. This difference may have been overlooked because previous studies employed animations created by a single graphic designer, or small group of experimenters and thus lack variation. However, this possibility demands empirical testing. Indeed, a direct comparison between our paradigm and previous studies is not possible due to task related differences (e.g., in indices of performance, and number of words animated per condition). If superior performance for non-mental, relative to mental, state animations were replicated, future studies may proceed to explore whether non-mental state videos are perhaps richer in cues (such as acceleration and ASFD in bin 6) which contribute to the correct inference of both mental- and non-mental states in the current dataset.

In this first analysis step it was further revealed that the triangles’ mean jerk in an animation plays a substantial role in interpreting that animation. For mental state attributions jerk was *negatively* predictive of accuracy, whereas for non-mental state animations jerk was *positively* predictive of accuracy. Post hoc analyses revealed that this latter result was primarily driven by fighting animations, and that the former was most notable with respect to mocking and surprising animations (though caution is advised with regards to the effect of surprising animations, since credible intervals of coefficient estimates did not exclude zero). In previous work, Edey and colleagues^9^ observed that non-autistic participants were more accurate in their mental state attributions for animations generated by non-autistic participants compared to those generated by autistic participants. They also observed that animations generated by autistic participants were more jerky compared to those generated by controls. However, in Edey et al.’s study there were a number of additional dimensions along which the two groups’ animations may have varied, making it impossible to know whether the autistic participants’ animations were difficult to interpret *because of* the jerky kinematics. Our results show that jerk meaningfully contributes to the accuracy of mental state attributions, thus our data supports the conclusion that jerk is highly likely to be one of the driving factors in the group differences observed by Edey et al.

Our results also highlight *jerk similarity* as a potential driving factor for the differences observed by Edey et al.^9^. That is, we observed a positive relationship between jerk similarity and accuracy. Post hoc analyses revealed that evidence of this relationship was particularly compelling in the case of mocking animations: The more closely a mocking animation’s mean jerk approximated the participant’s own jerk when animating the same word category, the more accurately that animation was rated. We speculate that Edey et al.’s non-autistic participants performed poorly when attributing mental states to animations produced by autistic individuals not only because these animations were jerky, but also because the kinematics of the animations were *dissimilar* from the way in which the observer would have produced the same animation. In other words, it is plausible that, in the minds of Edey et al.’s non-autistic observers, the animations generated by non-autistic animators triggered suitable mental state labels because the animation kinematics were similar to the kinematics that observers themselves would have produced. However, because the atypically jerky videos generated by autistic animators were presumably not, in the minds of non-autistic observers, associated with any mental state labels, it was difficult for observers to correctly identify the underlying mental state. This interpretation is in line with theoretical accounts^19–22,33^ suggesting that visual and/or motoric representations of one’s own actions may influence interpretations of others’ actions and is reminiscent of simulation accounts of theory of mind which claim that one uses one's own mental processes to simulate others’ mental states^23^.

The second aim of the current study was to explore the extent to which a range of other stimulus features, including trajectory shape, influence mental state attribution accuracy. To quantify trajectory shape we used FFT to decompose trajectories into spectral density in angular frequency bins. Animation identity could be differentiated by AFSD in nine bins and random forest analyses confirmed that four of these bins—bins 1, 6, 8 and 9 corresponding to angular frequencies 0.2–1.5, 4.8–5.2, 7.6–7.9, 8.8–10—were ‘important’ predictors of mental state attribution accuracy. Relative to the other words, following animations had the highest AFSD in the angular frequency range 0.2–1.5 (bin 1; Fig. 3). A high amount of AFSD in this range indicates a trajectory characterized by complex doodle-like movements (See Supplementary Fig. 3) with low angular-frequency oscillation in speed and curvature. Thus, one may speculate that animations which are most easily identifiable as ‘following’ comprise doodle-like triangle trajectories, with between 0.2 and 1.5 curvature oscillations per 2$\pi$ radians. In the angular frequency range 4.8–5.2 (bin 6), surprising animations had highest AFSD relative to the other words (See Fig. 3). This angular frequency range centers around the pure-frequency trajectory of a pentagon and thus is reflective of movements with around five speed-curvature oscillations per 2$\pi$ radians. Whilst our stimuli did not necessarily contain trajectories in the shape of actual pentagons, high AFSD in bin 6 reflects curves and speed-curvature patterns similar to those required to produce a closed-form pentagon. Finally, relative to the other words, both surprising and fighting had high AFSD in angular frequency ranges 7.6–7.9 (bin 8) and 8.8–10 (bin 9). A high amount of AFSD in these ranges indicates trajectories characterized by octagonal (bin 8) and decagonal shapes (bin 9) with 8–10 speed-curvature oscillations per rotation. Together these results clearly illustrate that trajectory shape comprises an important cue with respect to the identity of the word that is depicted in an animation. At present one can only speculate about why some shapes (e.g., pentagons) are more indicative of particular mental/non-mental states (e.g., surprising).

For the third step in our four-part analysis, we employed random forests to ascertain the relative contribution to accuracy of a range of stimulus features*.* The random forest methodology was chosen for its robustness against (multi-)collinearity and suitability for evaluating contributions of a large number of variables with limited data points^34^. Our random forest analysis confirmed ten features as important predictors of accuracy. In order of relative importance these are: mental state, mean rotation, acceleration, jerk, trajectory shape (AFSD in bins 1, 6, 8, 9), simultaneous movement of the triangles and speed. Post hoc analyses (see Supplementary Fig. 2) revealed that with respect to mental state attribution specifically, five of these features were of confirmed importance: jerk, acceleration, speed, AFSD-bin 6 and simultaneous movement. There was one feature which was uniquely important for mental state accuracy: The amount of simultaneous movement of blue and red triangles. By decomposing the animations task into features which predict accuracy, this random forest analysis deepens understanding of individual differences in animations task performance and raises testable empirical hypotheses for further research. For example, our analysis illustrates that simultaneous movement of the triangles is a stimulus feature which predicts mental state attribution accuracy. This observation raises the possibility that poor performance on the animations task in some clinical groups may be related to differences in processing this stimulus feature. That is, processing the simultaneous movement of the triangles requires distributed attention to two objects simultaneously. It may be that individuals with some clinical conditions (e.g., autism^35^) exhibit a deficit in the perception of global relative to local motion stimuli, making it more difficult for them to process the simultaneous movement of two triangles (though note contradictory evidence from Zwickel et al.^36^ that autistic participants distribute their attention evenly across both triangles). Given our finding that simultaneous movement cues are uniquely important for mental (but not non-mental) state accuracy, we speculate that difficulties processing the simultaneous movement of both triangles may impact selectively on the accuracy of mental-, not non-mental-, state attributions.

Finally, exploratory analysis 3 investigated whether similarity effects also exist with respect to other movement features such as triangle rotation. The random forest technique, due to its inability to account for random variance among individuals, does not allow for the inclusion of indices relating to movement similarity (which depend on the *relation between* an animation and an individual participant). We therefore conducted an additional set of Bayesian mixed effects models where we tested whether similarity between an observer and animator in the top three features identified by the random forest also predicted accuracy. Results revealed that, alongside jerk similarity, acceleration similarity and rotation similarity predict accuracy in the animations task. Whereas our *confirmatory analysis* was hypothesis-driven, this analysis illustrates that, even when a more data-driven approach is applied, jerk similarity is a strong predictor of accuracy. The results of this last exploratory analysis step are consistent with motor simulation accounts of mentalizing and extend the effect of jerk similarity to other movement features. It remains to be seen whether apparent mentalizing deficits in autism are ameliorated when autistic people are provided with stimuli which match closely to features of their own movement including mean rotation, trajectory shape and kinematics. If it were the case that movement similarity facilitates mental state attribution in clinical populations including autism and healthy people alike, it may be feasible to develop support systems to improve bi-directional communication between (for example) autistic and non-autistic people, by teaching counterparts to move more similarly in order to find a “common body language”. Although such support systems could be extremely helpful for autistic and non-autistic dyads who interact frequently, for instance in caregiver-caretaker relationships, there is much work that must yet be completed to build a bridge between the current findings and this future possibility.

This study has several limitations worth noting. First, with respect to generalizability, it should be noted that our 84% female sample lacked variation in terms of gender, age and educational background. In order to know whether our results apply to the wider population, future studies with more varied samples are needed. Second, since all of our participants first created their own animations *then* rated others’ animations, we cannot comment on whether the order of tasks influenced the results. The create-then-rate order was chosen to minimize the risk that, after *first* watching others’ animations, participants’ own movement kinematics would be biased towards the observed animations. However, we acknowledge that further studies are necessary to explore whether the jerk similarity effect here observed is weaker under rate-then-create conditions and/or when the delay between creating and rating animations is increased. Third, the current study demonstrates a role for jerk similarity in the accuracy of mental state attributions as indexed by performance on a Heider–Simmel style task but the extent to which these results extend to other ToM tasks is unknown. Although it is possible that movement similarity influences the accuracy of mental state attributions across a range of mentalizing tasks which require interpretation of body movement cues (e.g., the Movie for the Assessment of Social Cognition [MASC]-^37^ or the Silent Films^38^ task), this speculation must be confirmed with empirical testing. Finally, we note that our task does not allow inference of the direction of causality regarding movement jerkiness and mentalizing abilities. Movement jerkiness may precede mentalizing difficulties in some clinical conditions, whereas mentalizing difficulties emerge before motor symptoms in others (for detailed discussion see Eddy and Cook^39^). For instance, in autistic individuals, motor atypicalities have been noted from as early as one month of age^40^, whereas socio-cognitive differences tend to gradually emerge over the first few years of life^41^. Conversely, in Huntington’s disease, social cognitive symptoms have been found to occur before the onset of motor symptoms^42^. To clearly disentangle the direction of the relationship between socio-cognitive and motor development longitudinal studies that study these relationships within the same individuals, whilst accounting for potential mediating factors (see Happé et al.^43^, for further discussion about establishing cause and effect in socio-cognitive studies), are needed.

The present findings highlight particular kinematic- and trajectory features (specifically, acceleration, jerk, speed and energy in bin 6 (angular frequencies around 5); see Supplementary Fig. 3) as being important for mental state attribution in the context of the animations task. Based on our results one may speculate that individuals who experience difficulties with processing kinematic cues, or with trajectory tracking, may struggle to attend to, and/or process, the cues in Heider–Simmel style animations that are most relevant for accurate mental state attributions. That is, our results raise the possibility that individual differences in mentalizing may be related to individual differences in the perceptual processing of kinematics and trajectory information. Future studies which investigate the relation between kinematic processing, trajectory tracking, and mental state attribution accuracy are required to test this hypothesis. Our findings further show that similarity in a variety of movement features between observer and animator facilitates mental state attribution. Consequently, individuals with certain clinical conditions might find the animations task particularly difficult due to differences in perceptual processing and/or reduced movement similarity. Our data paves the way for studies which empirically test whether mentalizing deficits in clinical populations persist when participants are provided with stimuli which closely match features (including kinematics, trajectory shape and amount of simultaneous movement) of their own movements.

## Methods

### Building the animotions database

#### Animotion online task

We created a browser-based application that enables us to record and replay participants’ animations in the style of Heider and Simmel’s original movies^1^ while capturing the triangles’ positions at 133 Hz. For this purpose, we adapted a web application developed by Gordon and Roemmele (*The Heider–Simmel Interactive Theatre*^44^, https://hsit.ict.usc.edu/) to fit our task design and allow instantaneous calculation of mean speed, acceleration and jerk (change in acceleration), thus enabling the selection of stimuli according to predefined criteria for replay. Gordon’s web application employs scalable vector graphics (SVG) objects that allow simultaneous translation and rotation of each object with input from a single finger per object. To ensure object motion follows the direction of movement of the finger, and to suppress sporadic rotations (which can occur if dragging is initiated too close to the object center), object motion is suppressed until the pointer is dragged sufficiently far away from the center point (see https://asgordon.github.io/rotodrag-js/ for a more detailed description of the library used for this application).

#### Participants

We asked 51 healthy volunteers (46 females, mean (M) [SD] age = 20.23 [2.71] years, range 18–34 years) to animate two triangles in order to depict three mental state (mocking, seducing, surprising) and two non-mental state (following, fighting) words. Participants were recruited from the University of Birmingham research participation scheme, gave written informed consent and received either course credit or money (£8 per hour) for their participation. All experimental procedures were conducted in line with the WMA declaration of Helsinki^45^ and approved by the University of Birmingham Research Ethics Committee (ERN 16-0281AP5).

#### Procedure

Data was collected at the University of Birmingham. Individuals were seated in front of a WACOM Cintiq 22 HD touch screen, tilted at an angle of approximately 30 degrees upon the desk. They were presented with the starting frame, comprising a black rectangular enclosure and two equally sized equilateral triangles (one red and one blue) on a white background (see Supplementary Fig. 4). In a 45-s-long practice phase, participants familiarized themselves with how to use their finger movements in order to navigate the triangles around the screen. Participants were subsequently instructed to ‘*represent certain words by moving the triangles around the screen*’, assured they could move the triangles in any way they saw fit and encouraged to use their index fingers on both the left and right hand to move the triangles simultaneously (for a complete transcript of task instructions see Supplementary Materials). A dictionary was provided in case participants did not know the word in question. No further explanations were given.

Following instructions, participants were presented with the first word and a 30-s-long presentation of the stationary starting frame, allowing participants to plan their subsequent animation of that word. Finally, individuals were given 45 s to animate the given word. This process was repeated for the total of three mental state- (mocking, seducing, surprising) and two non-mental state words (following, fighting), and on each trial participants were given the option to discard and repeat their animations if they were unhappy with the result. Only the final animations were analyzed.

The mental state words were chosen to be the same as used in Edey et al.^9^, these words were based on the ToM animations used by Abell et al.^2^. After pilot testing revealed that participants found it difficult to understand the meaning of the word ‘coaxing’, even after consulting the dictionary, we removed this word. The non-mental state words were selected on the basis of the Goal-Directed animations used in Abell et al.

#### Stimulus selection

Our procedure resulted in a total of 255 animations (51 for each word), recorded at a frame rate of 133 frames/s. Animations were then visually inspected for sufficient length and movement coverage of more than two quadrants of the screen. 53 animations failed these quality control checks. The final stimulus set comprised 202 animations (42 mocking, 38 seducing, 36 surprising, 44 following, 42 fighting). Note that while our choice of words for the new animotions stimulus set was based on previous work by Abell et al.^2^ and Edey et al.^9^, none of their animation stimuli were used in this study.

### Ratings collection

#### Participants

Thirty-seven healthy volunteers (31 females, M [SD] age = 21.30 [2.68] years, range = 18–32 years) were recruited from the University of Birmingham Research Participation Scheme and gave written informed consent to participate in this study. Post-hoc power calculations based on an online application by Judd et al.^46^ (https://jakewestfall.shinyapps.io/two_factor_power/) confirmed that this study had 91.2% power to find an effect of size Cohen’s d (d) = 0.4 for the main hypothesis (1). An a priori power analysis of the complete study was not performed due to the lack of applications available to estimate effect sizes for the present analyses (a mixed effects model with more than one fixed effect). Participants received either course credit or money (£8 per hour) for their participation. None of the participants had previously taken part in stimulus development.

#### Task

**Figure 5:**
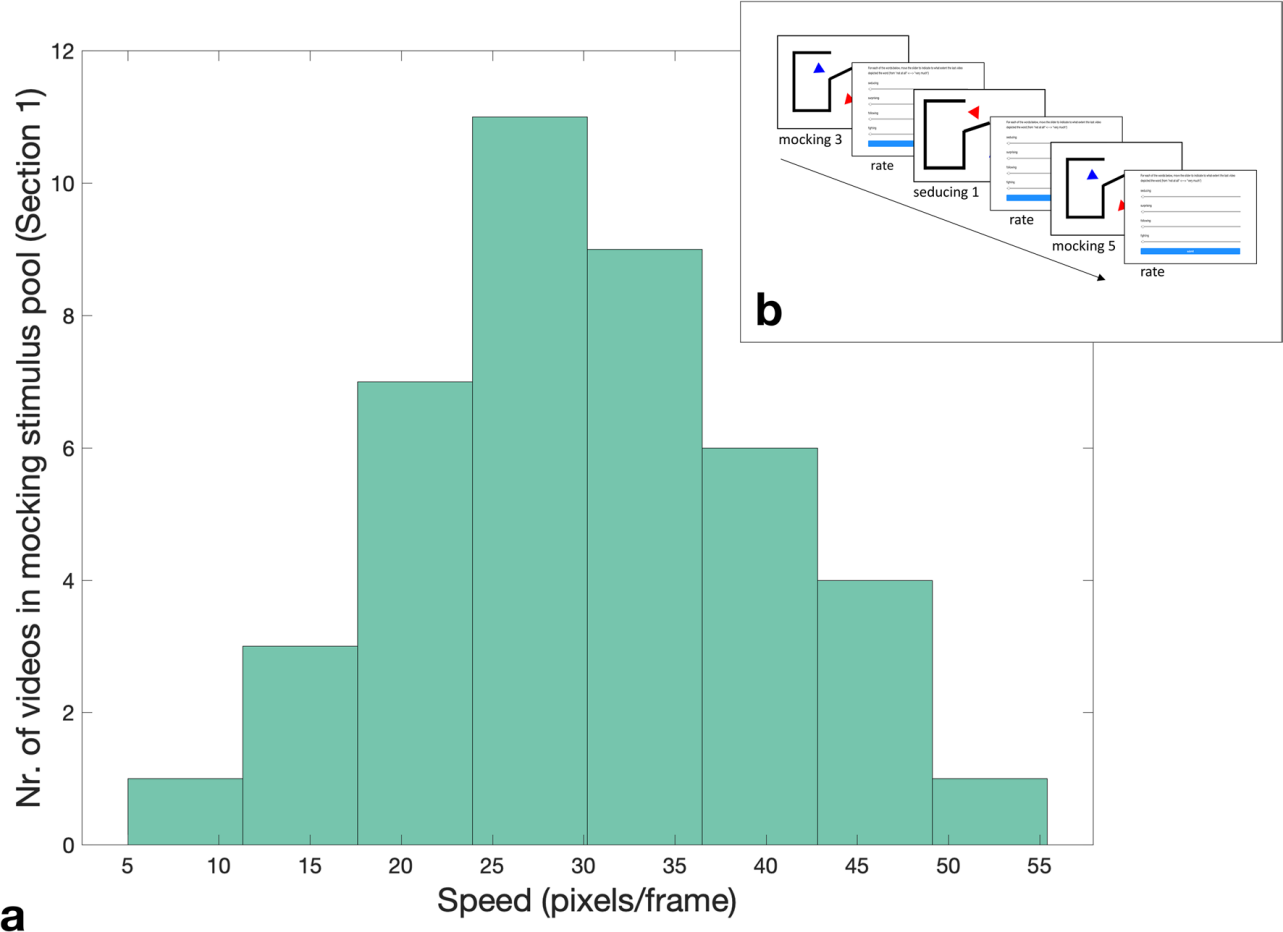
Example of stimulus selection method. (**a**) Example of the stimulus selection method for the word mocking. The selection method was the same for all five word categories. From each of eight percentile bins of the speed frequency distribution for a word category, one animation was selected at random and replayed to the participant. (**b**) Schematic depiction of 3 successive trials in the perception task. Numbers next to words represent the order number of the percentile bin from which the stimulus was selected (e.g., mocking 3 represents a mocking animation from the 3rd bin, which includes animations between the 25th and 37.5th percentile of the speed frequency distribution). Animations presented were selected at random; each animation was followed by a separate screen with five visual analogue sliding scales (one for each of the five word categories), ranging from 1 to 10.

The Ratings Collection phase comprised two tasks. First, all participants carried out a *production task*, where they created one 45-s-long animation for each of the five target words mocking, seducing, surprising, following and fighting, as described above. Following this, participants completed a perception task, where they viewed 40 animations from the full stimulus set and rated the extent to which the animations depicted each of the target words (mocking, seducing, surprising, following, fighting). Participants viewed eight exemplars of each of the five target words, presented in random order. The eight animations were selected from the stimulus pool (N = 202, see Building the animotions database) such that the mean speed of the triangles represented one of eight percentiles of the speed frequency distribution for a word (see Fig. 5). Thus, for each word, each participant viewed a selection of animations such that they were exposed to the full range of kinematic variation in the population used to create the stimulus pool.

Finally, after watching each animation, participants were asked to rate on a visual analogue scale ranging from one to ten the extent to which they perceived the video to display the target word (e.g., mocking) and each of the four non-target words (e.g., seducing, surprising, following and fighting). The whole process of creating five and viewing and rating 40–45 s animations lasted between 40 and 50 min. Task order was fixed (production task then perception task) to allow participants’ animations to be unaffected by the animations they would see in the perception task*.* Due to the upper limit on the WACOM monitor refresh rate, videos were created with a 133 Hz sampling rate and displayed at 60 Hz.

#### Procedure

Individuals sat in front of the WACOM Cintiq 22 HD touch screen, tilted at 30 degrees, and first completed a practice phase in which they familiarized themselves with moving the triangles around the screen. They were then instructed that they would first create an animation for each of the five words themselves (instructions were the same as in ‘Building the animotions database’; see Supplementary Materials) and subsequently would view and rate animations which had been created by other people. Participants then completed the production and perception tasks as described above.

### Data analysis and processing

All data was processed in MATLAB R2020a^47^ and analyzed in R^48^. Code required to reproduce data analysis and figures for this study will be freely available under (https://osf.io/pqn4u/).

#### Accuracy ratings

Accuracy for each trial was calculated by subtracting the mean rating for all non-target words from the rating for the target word. Thus, a positive score indicates that the target word was rated higher than all non-target words, with higher accuracy scores reflecting better discrimination between target and non-target words. See Appendix 1 for further analysis of accuracy scores.

#### Spatial and kinematic predictors

All variables were calculated from positional data derived from the center points of the blue and red triangles. All steps of data processing mentioned below were performed on both the animations created by participants (= production data) and the animations from the full stimulus set used as perception task stimuli (= perception data).

#### Stimulus kinematics

Instantaneous speed, acceleration magnitude and jerk magnitude were obtained by taking the first-, second- and third order non-null derivatives of the raw positional data, respectively (see [1], [2] and [3], where x and y represent x- and y positions of red and blue triangles in the cartesian coordinate system, $v$,$a$, and $j$ denote instantaneous velocity, acceleration and jerk, respectively, and $t$ denotes time).

1 $$\vec{v} = \sqrt {\left( {x_{t - 1} - x_{t} } \right)^{2} + \left( {y_{t - 1} - y_{t} } \right)^{2} }$$

2 $$\vec{a} = \frac{{d\vec{v}}}{{d\vec{t}}}$$

3 $$\vec{j} = \frac{{d\vec{a}}}{{d\vec{t}}}$$

As the ‘diff’ function in MATLAB amplifies the signal noise, which compounds for higher derivatives, we employed a smooth differential filter to undertake each step of differentiation (filter adopted from Huh and Sejnowski^24^). The Euclidean norms of the x and y vectors of velocity, acceleration and jerk were then calculated to give speed, acceleration magnitude and jerk magnitude. That is, speed is calculated as the distance in pixels moved from one frame to the next. Acceleration magnitude comprises the change in speed from one frame to the next, and jerk magnitude comprises the change in acceleration. Mean speed, mean acceleration magnitude and mean jerk magnitude were then calculated by taking the mean across red and blue values, respectively. Lastly, kinematic values were converted from units of pixels/frame to mm/s.

#### Observer-animator jerk similarity

In order to measure the similarity between participants’ and stimulus kinematic jerk, absolute observer-animator jerk difference was calculated by first subtracting the mean jerk of each video a person rated from their own jerk values when animating the same word, and then taking the absolute magnitude of those values. Lower jerk difference values indicate *higher* observer-animator jerk similarity.

#### Angular frequency spectral density (AFSD)

For the purpose of quantifying animation trajectories, we adapted a method developed by Huh and Sejnowski^24^. Huh and Sejnowski have shown that the two-thirds power law varies according to shape trajectory, such that the gradient of the relationship between angular speed and curvature (in the Frenet–Serret frame^49,50^) is a function of the shape’s angular frequency. Angular frequency here is defined as the number of curvature oscillations within one full tracing (360° or 2$\pi$ radians) of a closed-form shape. We extended the method to derive the angular frequencies of arbitrary trajectories (i.e., not closed-form shapes) from the frequencies of speed oscillations within every 2$\pi$ radians of a triangle’s angular displacement in the Frenet–Serret frame.

First, absolute instantaneous curvature k was calculated (angular speed divided by speed). This enables the calculation of Frenet–Serret speed *v*. Periodicity in *v*, in every 2$\pi$ radians, allows the determination of angular frequencies present in the triangles’ movement. Asymmetrical FFT was employed on log *v*, which returned the amplitude spectral density of all angular frequencies present for each triangle in each animation. Angular frequency values returned by the FFT were then interpolated to obtain uniformly sampled values at 1001 points.

**Figure 6:**
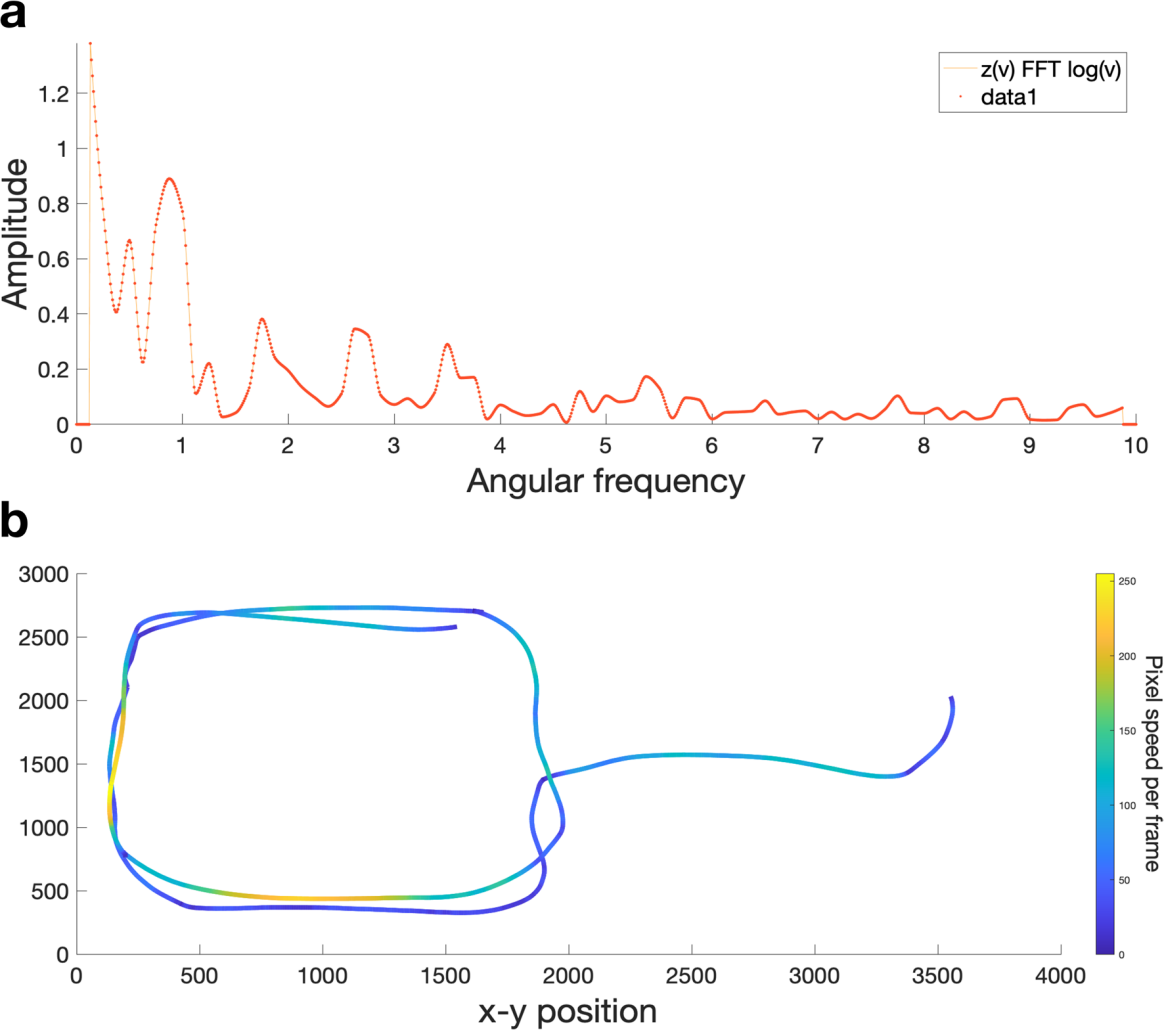
Example of trajectory shape and related angular frequency spectrum. (**a**) Example of angular frequency spectrum for following animation. (**b**) Related trajectory (of one of two triangles). Trajectory colors indicate speed (pixel/frame).

Because the FFT assumes an infinite signal, when addressing a finite sample such as the log angular speed here, the first and last values of each sample must be continuous to avoid artefacts in the FFT results. We addressed this and any general drift in the signal (e.g., from participants generally slowing their movements due to fatigue) by removing a second order polynominal trend. The area under the amplitude spectral density curve was normalized to allow like to like comparison between differing lengths of red and blue triangle movement within and across participants. Across red and blue triangles’ trajectories a weighted mean was then taken by multiplying each AFSD value with a factor reflecting the proportion of curved movement available for a triangle before averaging. See Fig. 6 for an example of an amplitude spectrum and the related trajectory path.

#### Further spatial variables

A variety of other variables were created to further quantify spatial aspects potentially affecting inferences from the animations. First, simultaneous movement was calculated as the proportion of all frames where both red and blue triangles’ speed was greater than zero (as seen in [4]), reflecting simultaneous movement of both triangles in a video. Furthermore, relative distance—the average distance between red and blue triangles—was quantified by taking the mean of the square root of the absolute distances between the triangles’ x and y coordinates, respectively (see [5]). Finally, mean rotation reflects the average rotation of blue and red triangles around their own axis, measured in angle degrees and weighted by proportion of movement present across all frames for each color [6].

4 $$\frac{{\sum \left( {speed_{red} \& speed_{blue} > 0.01} \right)}}{\sum all\;frames}$$

5 $$\underline {x} \left( {\sqrt {\left( {abs\left( {x_{red} - x_{blue} } \right)} \right)^{2} + \left( {abs\left( {y_{red} - y_{blue} } \right)} \right)^{2} } } \right)$$

6 $$\frac{{\left( {\underline {x} \left( {abs\left( {r_{blue t - 1} - r_{blue t} } \right)} \right)} \right) + \left( {\underline {x} \left( {abs\left( {r_{red t - 1} - r_{red t} } \right)} \right)} \right)}}{2}$$

### Statistical analysis

#### Data analysis overview

This study investigates the role of a large number of different predictor variables in explaining accuracy in the animations task. For two of these variables we present specific hypotheses (jerk, jerk difference); in addition, we wanted to investigate the role of a larger set of variables on an exploratory basis. For this reason, analyses were conducted in two stages: First, in a confirmatory stage, the roles of jerk and jerk difference were examined using Bayesian mixed models. Second, in an exploratory stage, a random forest model was performed, investigating the relative contribution of all predictor variables together.

#### Data cleaning and transformations

For all analyses, variables that were not normally distributed were either log- or square-root transformed to approximate normal distribution. Any outliers, as defined by values exceeding three scaled absolute deviations from the median, were replaced with the respective lower and upper threshold values. Finally, all predictor variables were z-scored.

#### Confirmatory analysis

A Bayesian linear mixed effects model was fitted in R using the *brms* package^51^ to evaluate the relative contribution of jerk and jerk difference to accuracy as a function of word category, as well as their three-way interaction. A maximal^25^ random effects structure was defined, allowing the intercept, the predictors of interest and their interactions to vary by participants (subject ID) and items (animation ID). Jerk and jerk difference were entered as covariates and word category was entered as dummy coded factor. We used brms default priors for the intercept and the standard deviation of the likelihood function as well as weakly informative priors (following a normal distribution centered at 0 and SD = 10) for all regression coefficients. Each model was run for four sampling chains with 5000 iterations each (including 1000 warmup iterations). There was no indication of convergence issues for any of the models (all Rhat values = 1.00, no divergent transitions).

#### Exploratory analysis 1

Bootstrapped F-tests were performed to test for differences, between the five target words, in the presence of angular frequencies at each of the 1001 points on the amplitude spectrum. Bootstrapping amplitude spectrum values 1000 times revealed nine significant clusters, defined as clusters of difference that occurred in less than 5% of comparisons with resampled distributions (see Fig. 3a). The maxima and minima of each significant cluster were then used as bin edges for calculating the amplitude spectral density as the area under the curve within those nine bins, for both red and blue triangles’ trajectories in each animation (cluster bin edges: 0.21–1.49, 1.61–2.39, 2.64–2.87, 3.04–3.40, 3.91–4.27, 4.79–5.19, 6.19–6.68, 7.6–7.93, 8.75–10). Finally, the weighted mean (weighted by amount of curved movement present in a triangle’s full trajectory) was taken across red and blue triangles’ spectral density values to form a single value of mean AFSD for each of nine bins for each animation. The final spectral density values are reflective of the relative proportion of curved movement available in a video in each of the nine areas of interest. Thus, a video that had high spectral density in bin 3 would be dominated by shapes with angular frequencies between 2.64 and 2.87. That is, relative to all other animations, the triangles in this video would be predominately moving with a speed and acceleration profile that lies between that of elliptical- and triangle trajectories.

#### Exploratory analysis 2

Relative variable importance of 16 variables in predicting accuracy was assessed using random forest models^28^ and the feature selection wrapper algorithm *Boruta*^29^. Random forests are ensembles of decision trees, where each tree is grown from a pre-specified subset of bootstrapped samples and where, for each tree, only a randomly selected subset of variables are considered as splitting variables. Boruta makes use of the *ranger* package^52^ to train a random forest regression model on all variables as well as their permuted copies—so called “shadow features”. First, *normalized permutation importance* (scaled by standard error, see^28^) of all features is assessed. Permutation importance of a given variable is the reduction in prediction accuracy (mean decrease in accuracy, MDA) of the model when this variable is randomly permuted. A variable is then classed as important when the Z-score of their importance measure is significantly higher than the highest importance Z-score achieved by a shadow feature. Overall performance of the model was evaluated by fitting a random forest with the ranger package with 500 trees and 10 random variables per tree.

Due to the known correlational structure within the data and the present lack of random forest models which can account for random effects, this analysis was performed items-based. For this purpose, for every variable, values corresponding to the same item were averaged across subjects, resulting in a total of 202 data points. Note that, due to the reliance on between-subject variance of variables relating to own-stimulus kinematic difference, these variables were excluded from this analysis.

#### Exploratory analysis 3

*Acceleration difference* and *rotation difference* were calculated as the difference in acceleration and mean rotation between an animation stimulus and the participant’s own animation of the same word, where lower difference values indicate higher movement similarity. Subsequently, we used Bayesian mixed effects models, with the maximal possible random effects structure, to quantify the strength of these difference scores in predicting mental state attribution accuracy. However, high variance inflation factors (VIFs) for the predictors jerk difference (VIF = 33.31) and acceleration difference (VIF = 33.84) indicated collinearity. To avoid the problem of inflated standard errors associated with high VIFs^53^, we used two mixed effects models: the first, with acceleration difference, rotation difference and mental state predicting accuracy, and the second with the predictors rotation difference, jerk difference and mental state.

## Supplementary Information

Supplementary Information.
