# Supplementary material for: Kinematics and observer-animator kinematic similarity predict mental state attribution from Heider–Simmel style animations
Source: Sci Rep. 2021 Sep 14;11:18266. doi: 10.1038/s41598-021-97660-2 (PMC8440512; doi:10.1038/s41598-021-97660-2)
Supplement: Supplementary file 1 — Supplementary Information. [file 41598_2021_97660_MOESM1_ESM.pdf]

Supplementary Information

Kinematics and observer-animator kinematic similarity predict mental state attribution from Heider-Simmel style animations

Bianca A. Schuster, Dagmar S. Fraser, Jasper J.F. van den Bosch, Sophie Sowden, Andrew S. Gordon, Dongsung Huh, and Jennifer L. Cook

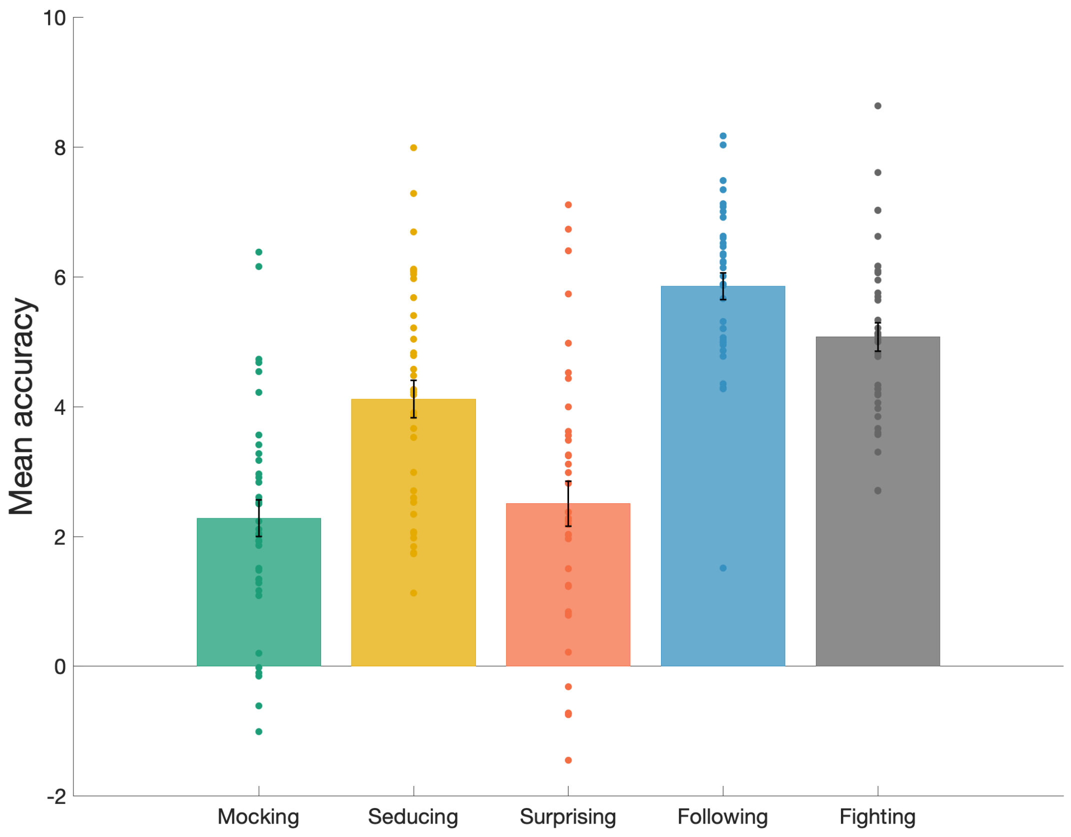

Supplementary Figure 1. Mean Accuracy Ratings for the Five Word Categories. Error bars represent 1 standard error of the mean (SEM).

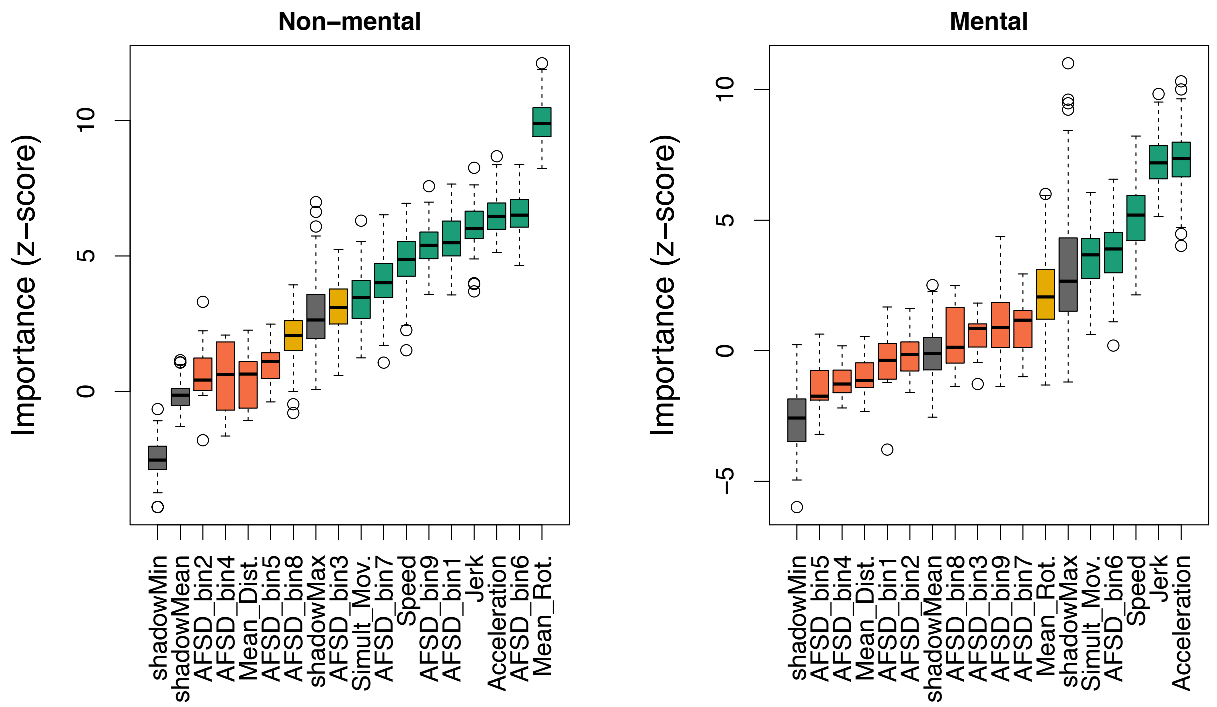

Supplementary Figure 2. Post-hoc random forest models. Box edges denote the interquartile range (IQR) between first and third quartile; whiskers denote 1.5 * IQR distance from box edges; circles represent outliers outside of 1.5 * IQR above and below box edges. Box color denotes decision: Green = confirmed, yellow = tentative, red = rejected; grey = meta- attributes shadowMin, shadowMax and shadowMean (minimum, maximum and mean variable importance attained by a shadow feature).

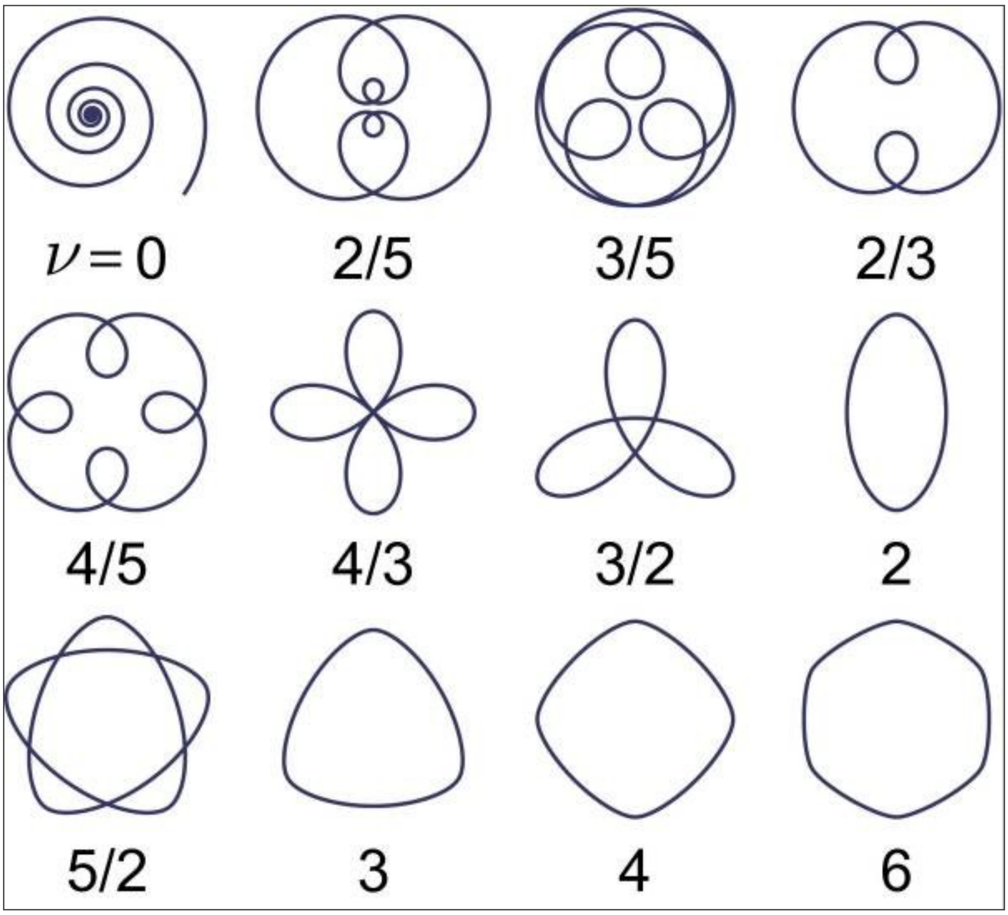

Supplementary Figure 3. Examples of pure frequency shapes shown with their characteristic frequencies. Figure taken with permission from Huh & Sejnowski (2015). See https://www.youtube.com/watch?v=waXWOv0YqFE for a movie showing how the shape of the curve varies with continuously changing angular frequency.

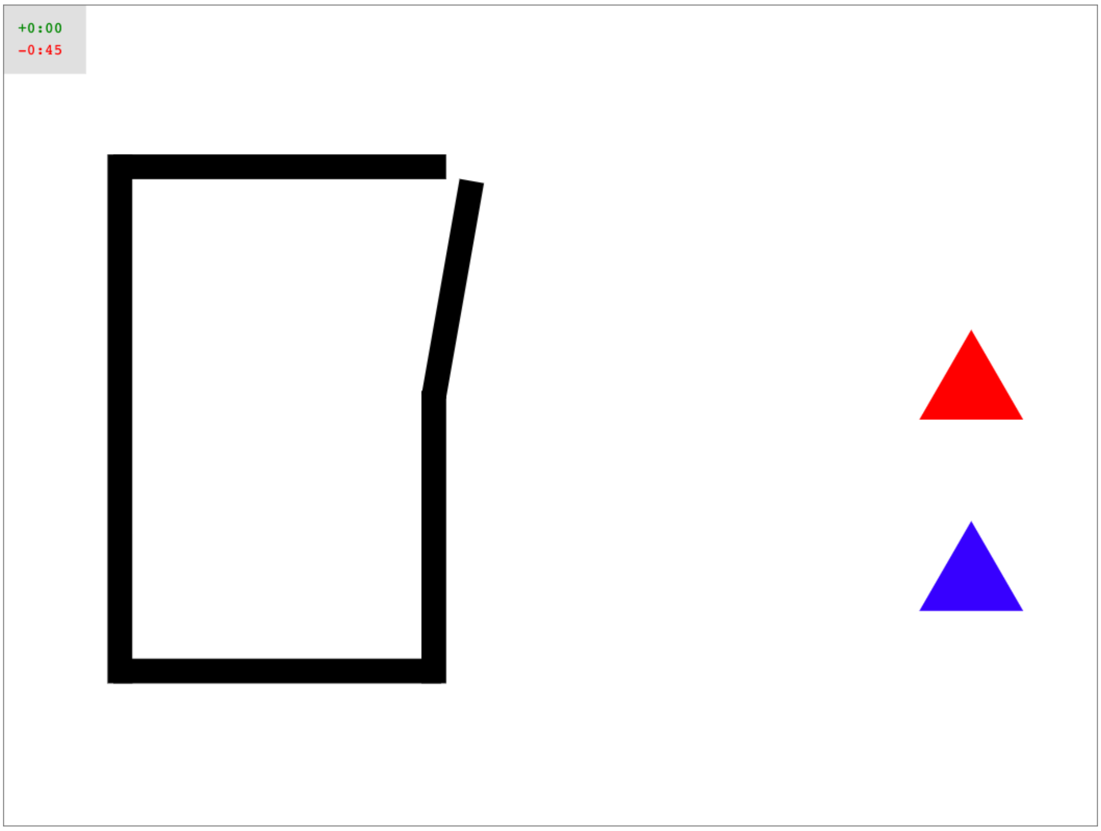

Supplementary Figure 4. Animotions task starting screen.

Appendix 1

Further analysis of accuracy scores

A one-way analysis of variance (ANOVA) comparing rating accuracy in the five word categories showed a main effect of word category, indicating that the five types of animations differed with respect to how accurately they were rated ($F(4,175) = 31.03$, $p < .001$). Separate Bonferroni-corrected post-hoc t-tests (using the Matlab *multcompare* function with Bonferroni correction) revealed that while there was no difference in accuracy between the words following and fighting (following mean (M) = 5.86, standard error of the mean (SEM) = 0.28, fighting M = 5.07, SEM = 0.22, $p = .507$), both words were rated with higher accuracy than mocking (mocking M = 2.28, SEM = 0.28, both $p < .001$), and surprising (surprising M = 2.51, SEM = 0.35, both $p < .001$) and following was rated more accurately than seducing (seducing M = 4.12, SEM = 0.29, $p < .001$). Furthermore, seducing animations exhibited higher accuracy than both mocking and surprising videos (both comparisons $p < .001$), whereas there was no difference in accuracy between mocking and surprising ($p = 1.000$).

Appendix 2

Task instructions

Production task

In the following task you will be asked to use your fingers to move two triangles around the screen in order to depict various words. Both triangles can be moved at the same time using both of your index fingers. After you press continue you will have a chance to practice moving the triangles around.

Perception task

The main task will now begin. On the screen you will be presented with a word. Please move the triangles around to represent the word on the screen. You will be presented with five words in total, one after the other. You can move the triangles around in any way you want, as long as you think their movements represent the word. Remember that you can move both triangles at once!

After you press 'continue', the first word will appear on the screen, along with a timer. You

will have 30 seconds to plan how you are going to move the triangles to depict the word. You will not be able to move the triangles during the 30 second thinking time. If you don't know the meaning of a word, you can look it up in the dictionary during this time.

When the thinking time is up, you will have 45 seconds to create your animation. Please try and use all of that time to create your story. If you are happy with your animation, press 'submit' and you will be presented with the next word. If for any reason you are not happy with your animation, you can press 'start over' and restart that word as many times as you like.
